# Role of bilirubin in the prognosis of coronary artery disease and its relationship with cardiovascular risk factors: a meta-analysis

**DOI:** 10.1186/s12872-022-02899-w

**Published:** 2022-11-02

**Authors:** Xiao-ling Li, Cun-rui Zhao, Chen-liang Pan, Gaxue Jiang, Bo Zhang

**Affiliations:** 1 Department of Neurology, Lanzhou University Second Hospital, Lanzhou, 730030 Gansu China; 2 Department of Cardiology, The First Hospital of Lanzhou University, No.1, Donggang West Road, Lanzhou, 730013 Gansu China

**Keywords:** Coronary heart disease, Bilirubin, Prognosis

## Abstract

**Background:**

Bilirubin is a heme catabolism product with antioxidant, anti-inflammatory, and anti-apoptotic properties and is implicated in the prognosis of several diseases. This study evaluates the prognostic role of bilirubin in coronary artery disease (CAD) patients.

**Methods:**

After identifying studies from the literature, meta-analyses were performed to achieve a) overall estimates of serum total bilirubin levels in patients with myocardial infarction (MI), non-MI CAD and healthy individuals; b) odds ratios (OR) of adverse outcomes between higher and lower total bilirubin levels; c) standardized mean difference (SMD) in total bilirubin levels in patients with high vs low CAD severity; and d) correlation between disease severity and total bilirubin. Metaregression analyses were performed to examine the relationship between cardiovascular risk factors and increasing quantiles of total bilirubin levels.

**Results:**

Forty-three studies were identified. Pooled serum total bilirubin levels were 0.72 mg/dl [95% confidence interval (CI): 0.60, 0.83] in MI patients; 0.65 mg/dl [95% CI: 0.60, 0.69] in non-MI CAD patients; and 0.66 mg/dl [95% CI: 0.56, 0.75] in healthy individuals. Higher total bilirubin levels were associated with greater odds of adverse outcomes in MI patients (OR: 1.08 [95% CI: 0.99, 1.18]) but lower odds in non-MI CAD patients (OR: 0.80 [95%CI: 0.73, 0.88]). Compared to non-severe cases, total bilirubin levels were higher in patients with severe MI (SMD 0.96 [95% CI: − 0.10, 2.01]; *p* = 0.074) but were lower in severe non-MI CAD patients (SMD − 0.30 [95%CI: − 0.56, − 0.03]; *p* = 0.02). Total bilirubin levels correlated positively with MI severity (*r* = 0.41 [95% CI: 0.24, 0.59]; *p* < 0.01) but correlated negatively with non-MI CAD severity (*r* =  − 0.17 [95% CI: − 0.48, 0.14]; *p* = 0.28). Female sex was inversely associated with increasing quantiles of bilirubin (meta-regression coefficient: − 8.164 [− 14.531, − 1.769]; *p* = 0.016) in MI patients.

**Conclusion:**

Prognostic role of bilirubin for CAD appears complicated, as different odds are observed for MI and non-MI CAD patients which weakens the case of causal involvement of bilirubin in CAD etiology or prognosis.

**Supplementary Information:**

The online version contains supplementary material available at 10.1186/s12872-022-02899-w.

## Introduction

Coronary artery disease (CAD) is the most common heart disease. It is an atherosclerotic disease in which blood flow is impeded in the coronary arteries due to the formation and deposition of plaque on the vessel wall that can lead to angina, myocardial infarction (MI), or sudden death [1–3]. The prevalence of CAD in the general population is estimated to be 6.3% and is less in women (5.2%) than in men (7.5%) [4]. The correlation between the prevalence of CAD and the human development index is negative in developed countries (*r* =  − 0.34) but positive in developing countries (*r* = 0.47) [4]. CAD burden is increasing in low-income and middle-income countries [5]. However, low education and socioeconomic status increase the odds of CAD even in developed countries [6]. Risk factors for the development of CAD include later age, male sex, diabetes mellitus, hypertension, smoking, dyslipidemia, obesity, homocystinuria, renal dysfunction, sedentary lifestyle, unhealthy diet, and family history/genetics [3, 7–11].

Bilirubin is a heme degradation product. During the catabolism of hemoglobin, heme is converted into biliverdin by heme oxygenase which is acted upon by biliverdin reductase to form bilirubin. In liver cells, bilirubin is changed to a conjugated form for secretion in bile juice [12]. At concentrations found in human plasma, bilirubin acts as an antioxidant to scavenge peroxyl radicals as efficiently as alpha-tocopherol [13, 14]. Both free and bound forms of bilirubin can inhibit the oxidation of low-density lipoproteins at physiological concentrations [15, 16]. Oxidation of low-density lipoproteins is an important initial step in atherogenesis that can stimulate platelet aggregation and can alter vasomotor properties [14]. Gilbert's syndrome patients have a reduced risk of cardiovascular disease (CVD) which is associated with increased bilirubin levels and altered lipid and inflammatory profiles [17]. At pathological levels, unconjugated bilirubin inhibits cytotoxic T cell activity and proliferative responses to human peripheral blood mononuclear cells in patients with neonatal or obstructive jaundice [18].

Research conducted during the last few decades has revealed that bilirubin may have a role in the prognosis of CAD. An inverse relationship was reported between serum bilirubin levels and atherosclerosis [19] and higher serum bilirubin levels were found to be associated with a better prognosis in patients with arteriosclerotic diseases [20]. Another meta-analysis found a U-shaped curve to depict the relationship between bilirubin and CVD [21] However, whereas elevated total bilirubin levels (within physiological limits) are reported to be associated with a lower risk of first MI [22], in patients with MI, a positive relationship is found between serum bilirubin levels and major adverse cardiovascular events (MACE) [23]. These observations provide an impetus for a review of studies that sought the associations between bilirubin levels and the prognosis of CAD. The objective of the present study was to conduct a systematic review of studies that investigated the bilirubin levels in CAD patients and sought the associations between bilirubin levels and disease severity in MI and non-MI CAD patients and to perform meta-analyses of important statistical indices.

## Methods

The present study was performed by following PRISMA guidelines [24].

### Eligibility

Inclusion criteria were: a study recruited patients with CAD to evaluate the prognostic role of bilirubin in MI and non-MI CAD patients and reported (a) bilirubin levels; (b) odds ratios of adverse outcomes between higher and lower bilirubin levels; and (c) correlations between bilirubin levels and disease severity. Studies were excluded if reported (a) the associations between bilirubin and non-cardiovascular complications; (b) non-prognostic associations; (c) associational point estimate/s other than odds ratios; and (d) the changes in bilirubin levels after MI or coronary intervention.

### Literature search

A literature survey was conducted in the Google Scholar, PubMed, Ovid, and Science Direct databases using the most relevant keywords including coronary artery disease, CAD, coronary heart disease, CHD, acute coronary syndrome, ACS, ischemic heart disease, IHD, angina, myocardial infarction, MI, percutaneous coronary intervention, PCI, bilirubin, prognosis, prognostic, association, and correlation. Literature search strategy is presented in Appendix S1. References lists of important research and review articles were also screened. The literature search encompassed original articles published in English from the date of database inception until March 2022. Two reviewers screened the records independently and then merged their listings. Study selection was carried out with mutual consultations and if any disagreement appeared, a third reviewer was involved.

### Data analyses

Demographic and clinical data, prevalent conditions, medications, lipid profiles, liver function data, CVD risk factor data, and statistical endpoints were extracted from the research articles of respective eligible studies and were organized on data sheets. Two reviewers extracted data from research articles independently and then unified output with mutual consultation. Quality assessment of the included studies was performed with Newcastle–Ottawa Scale for Observational Studies [25] and publication bias assessment was performed with Begg’s rank correlation test [26].

Total bilirubin levels reported by the individual studies were pooled using the DerSimonian-Laird method in patients with MI and non-MI CAD and in healthy individuals. Random-effects meta-analyses of standardized mean difference (SMD) was performed to assess the significance of difference in total bilirubin levels in patients with high vs low CAD severity. Odds ratios (ORs) of adverse outcomes between higher and lower total bilirubin levels in CAD patients reported by the individual studies were pooled using the DerSimonian-Laird method [27]. Subgroup analyses were performed by categorizing MI and non-MI CAD patients.

A meta-analysis of correlation coefficients was performed under the random-effects model to estimate the correlation between total bilirubin levels and disease severity. For this meta-analysis, correlation coefficient values were first converted to Fisher’s z-scores and their respective standard errors were derived from the sample sizes. Meta-analysis outcomes were back-transformed to correlation coefficients.

To seek linear relationships between the increasing quantiles of total bilirubin and several explanatory variables, meta-regression analyses were performed using the restricted maximum likelihood method. Explanatory variables were the age, sex, body mass index, hypertension, dyslipidemia, diabetes mellitus, smoking, CVD history, total cholesterol, triglycerides, high-density lipoprotein, low-density lipoprotein, hemoglobin, high-sensitivity c-reactive protein, serum creatinine, alanine transaminase, aspartate transaminase, gamma-glutaryl transaminase, alkaline phosphatase, statin use, angiotensin-converting enzyme inhibitors/angiotensin receptor blocker use, calcium channel blocker use, and beta-blockers use.

The statistical index used to estimate heterogeneity was I^2^ which assesses between-study inconsistency in the outcomes. It informs what proportion of the observed variance reflects differences in true effect sizes rather than sampling error [28]. Begg’s rank correlation test was performed for the assessment of publication bias. All statistical analyses were performed with Stata software (version 12; Stata Corporation, College Station, Texas, USA).

## Results

**Fig. 1:**
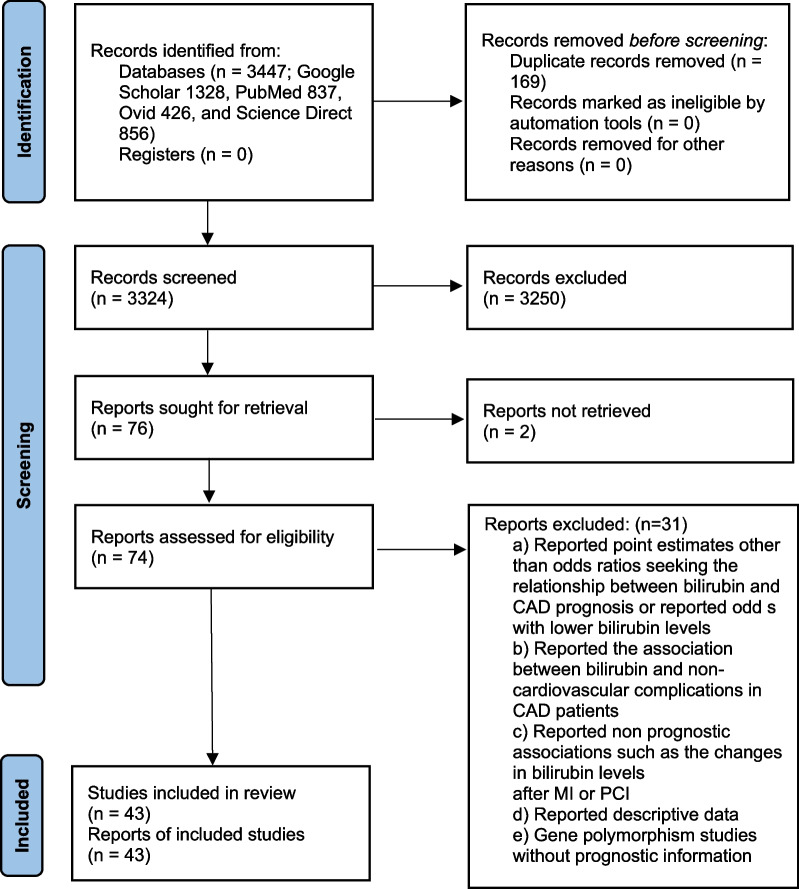
A flowchart of the study screening and selection process

Forty-three studies [29–71] were included (Fig. 1). In these studies, 34,976 patients with CAD and 29,229 non-CAD individuals from general populations were recruited. The important characteristics of the included studies are presented in Table 1. There was no significant publication bias according to the Begg’s test (adjusted Kendall’s score: − 20 ± 86; *p* = 0.816; Figure S1). The quality of the included studies was generally moderate to high (Table S1).

**Table 1:** Characteristics of the included studies

Study	n	Design	Patients	Age (years)	% Females	BMI	% HTN	% DL	% DM	% Smokers	% CVD history
Acet 2014 [29]	360	RET	STEMI	61 ± 14	26.5		36	5	24	56	5
Agarwal 2015 [30]	319	CS	CAD	57 ± 10	23.67	27.5 ± 3.1	30		28	43	
Akboga 2015 [31]	1121/380	RET	Angina/HI								
Baumann 2016 [32]	803	RET	STEMI	63 ± 13			59	30	24	50	17
Canpolat 2013 [33]	1115	RET	CAD	59 ± 12	55.575	27.8 ± 4.3	64	56	54	37	15
Celik 2014 [34]	536	RET	STEMI	60 ± 12	22.5	26.2 ± 2.6	43		26	54	21
Chung 2016 [35]	1111	RET	STEMI	62 ± 13	23.5	23.5 ± 3	40	23	30	49	5
Ekblom 2010 [36]	618/1184	PROSP	MI/HI	59 ± 14		27.2 ± 1	46				
Elmohr 2021 [37]	90	RET	STEMI	57 ± 12	24.5		34	52	33	53	
Erkan 2014 [38]	299/76	RET	CAD/HI	65 ± 10	17.4		72	55	40	48	
Gul 2013 [39]	1624	RET	STEMI	57 ± 12	15		39	37	25	58	12
Gullu 2005 [40]	160	PROSP	HI	37 ± 7	53.80	25.7 ± 3.3					
Hamur 2016 [41]	229	RET	STEMI	63 ± 8	27.5	25.8 ± 1.2	34	42	30	42	13
Hopkins 1996 [42]	161	RET	MI	56 ± 6	25.5	28.7 ± 4.7			14	52	
Huang 2017 [43]	3103	RET	CAD	64 ± 11	20.5	24.3 ± 3.3	55		22		13
Hunt 2001 [44]	148/180	PROSP	CAD/HI	62 ± 7	33.11	28.7 ± 5.3				11	
Kaya 2014 [45]	403	RET	NSTEMI	65 ± 12	34	26.5 ± 3	50		33	34	
Khalil 2019 [46]	70	PROSP	STEMI				57		69	49	
Kim 2015 [47]	372	RET	CAD	65 ± 11	37		57	26	33	48	
Kishimoto 2020 [48]	124/138	RET	CAD	66 ± 12	40	24.2 ± 4.8	60	37	10	35	
Kuwano 2011 [49]	1076	RET	CAD	66 ± 11	17.3	23.6 ± 3.1	76	72	48	33	32
Lai 2018 [50]	12,097	Cohort	HI	63 ± 8	54.4	24 ± 3.2	49	49	16	19	
Mahabadi 2014 [51]	3553	Cohort	HI	59 ± 8	56.5	27.8 ± 4.6	32		12	32	
Miranda 2016 [52]	145	RET	STEMI	61 ± 13	16	27.5 ± 3.5	56	48	15	52	9
Oda 2012 [53]	5444	Cohort	HI	56 ± 10		22.7 ± 3				17	
Sahin 2013 [54]	281	RET	STEMI	61 ± 12	33.5	26.5 ± 2.6				61	
Schwertner 1994 [55]	619	RET	CAD	42 ± 7						30	
Song 2014 [56]	8593	PROSP	CAD/HI	52 ± 9	47	24.4 ± 3	21		14	25	
Tanaka 2009 [57]	637	RET	CAD	69 ± 11	38	23.9 ± 4	16			33	
Tatami 2014 [58]	394	RET	CAD	69 ± 9	20	23.6 ± 3.9	77	81	42	25	21
Troughton 2007 [59]	335/670	RET	CAD/HI			27.3 ± 3.6					
Turfan 2013 [60]	299	RET	CAD	61 ± 12	46.7		30		27	27	
Tuxun 2020 [61]	615	RET	STEMI	58 ± 12	14	25 ± 3.1					
Wang 2017 [62]	2478	PROSP	DM	64 ± 8	54		39		100	26	
Wei 2012 [63]	1280	RET	Angina	59 ± 8	40.2		59		20		
Xu 2018 [64]	405	RET	MI	61 ± 13	14		57	8	21	67	
Xu 2019 [65]	533	RET	CAD	60 ± 10	22	25 ± 3.3	56		24	56	
Yao 2015 [66]	1419	RET	Angina	61 ± 11	34.5	24 ± 4	54	49	21	32	21
Yu 2017 [67]	347	RET	CAD	64 ± 11	35.1585		68	49	34	27	
Zhang 2012 [68]	3408	RET	STEMI				37		12	29	
Zhao 2020 [69]	4151	RET	STEMI	58 ± 12	15		60	93	32	69	
Zhu 2012 [70]	638	RET	CAD	63 ± 10	50	23.7 ± 1.7			23	40	
Zhu 2016 [71]	**130**	**PROSP**	**CAD/HI**	**63 ± 10**	**33.25**	**25.9 ± 3.8**	**53**		**19**	**32**	

*Abbreviations:* *BMI* Body mass index, *CAD* Coronary artery disease, *CS* Cross-sectional, *DL* Dyslipidemia, *DM* Diabetes mellitus, *HI* Healthy individuals, *HTN* Hypertension, *PROSP* Prospective, *RET* Retrospective, *MI* Myocardial infarction, *STEMI* ST-segment elevation MI

**Fig. 2:**
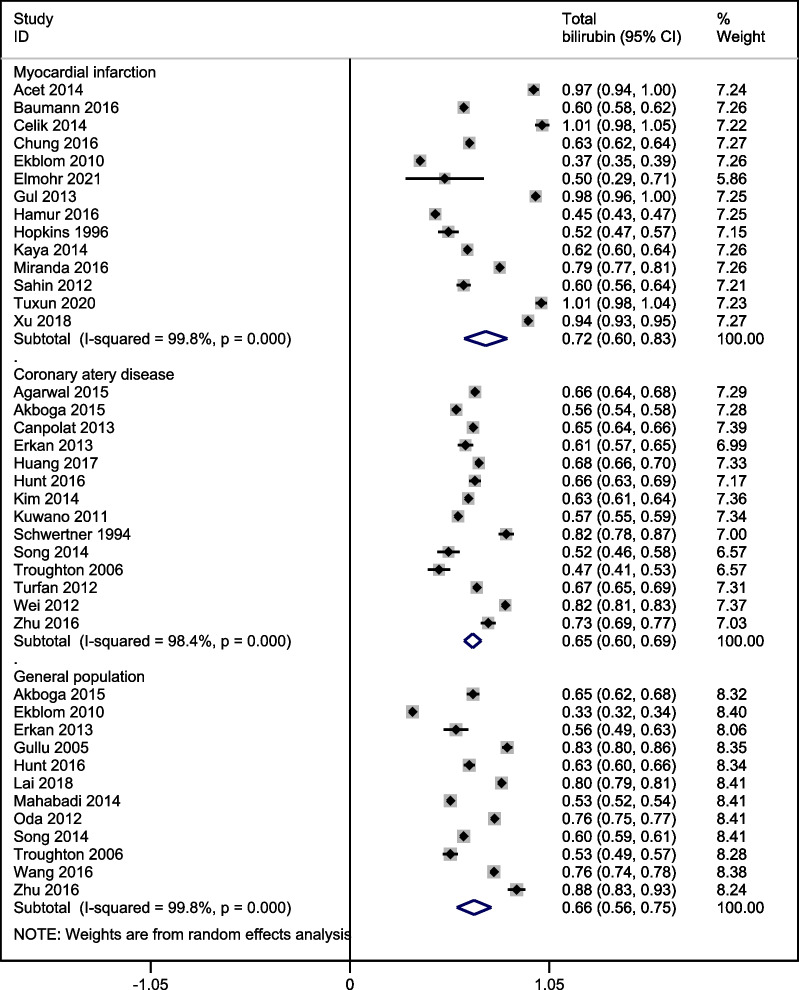
A forest graph showing the pooled estimates of serum total bilirubin levels in MI patients, non-MI CAD patients, and individuals from the general population

In the pooled analysis, serum total bilirubin levels were 0.72 mg/dl [95% confidence interval (CI): 0.60, 0.83] in MI patients; 0.65 mg/dl [95% CI: 0.60, 0.69] in non-MI CAD patients; and 0.66 mg/dl [95% CI: 0.56, 0.75] in healthy individuals from general populations (Fig. 2).

**Fig. 3:**
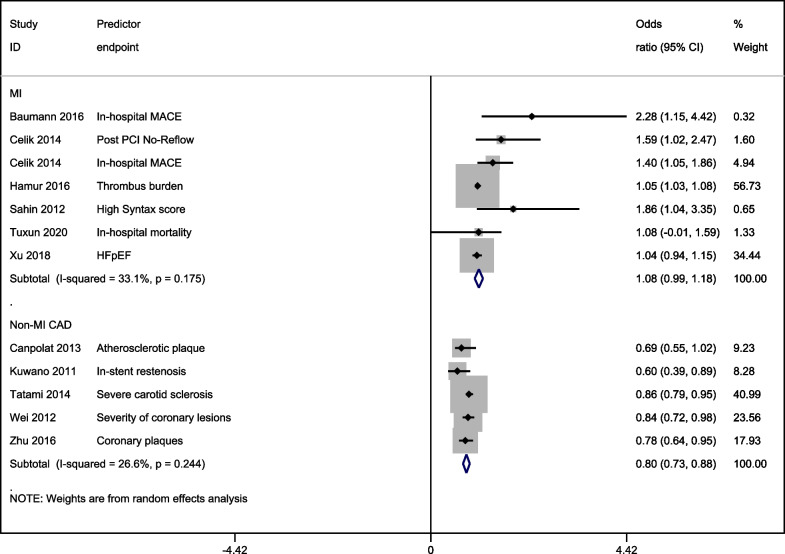
A forest graph showing the pooled estimates of odds ratios of adverse outcomes between higher and lower bilirubin levels in MI and non-MI CAD patients. Abbreviations: HFpEF, Heart failure with preserved ejection fraction; MACE, major adverse cardiovascular events; PCI, percutaneous coronary intervention

A pooled analysis of the odds ratios reported by the individual studies found higher odds of adverse outcomes with higher total bilirubin levels in MI patients (OR: 1.08 [95% CI: 0.99, 1.18]) but lower odds with higher total bilirubin levels in non-MI CAD patients (OR: 0.80 [95% CI: 0.73, 0.88]; Fig. 3).

**Fig. 4:**
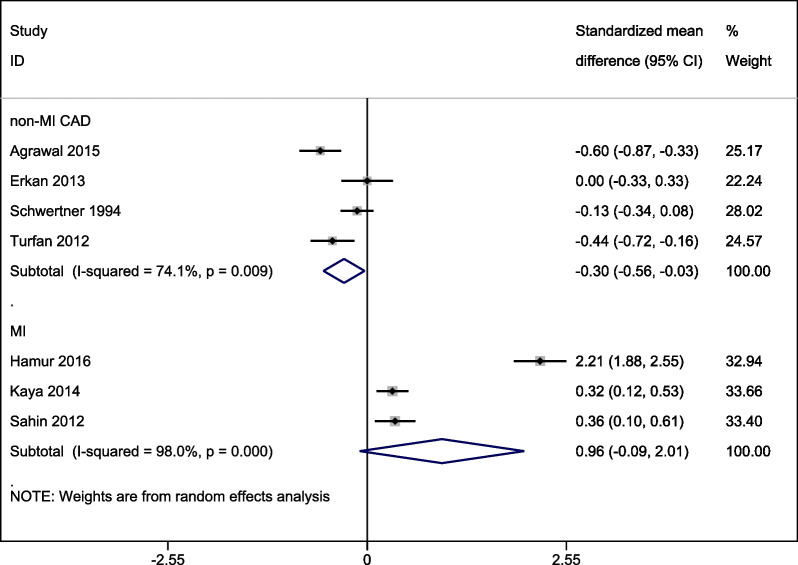
A forest graph showing the outcomes of a meta-analysis of standardized mean difference (SMD) in bilirubin levels between higher and lower disease severity indices

A meta-analysis of SMD in bilirubin levels between higher and lower disease severity indices found statistically non-significantly higher total bilirubin levels in patients with severe MI (SMD 0.96 [95% CI: − 0.095, 2.01]; *p* = 0.074) but significantly lower total bilirubin levels in patients with severe non-MI CAD (SMD − 0.30 [− 0.56, − 0.03]; *p* = 0.02; Fig. 4).

**Fig. 5:**
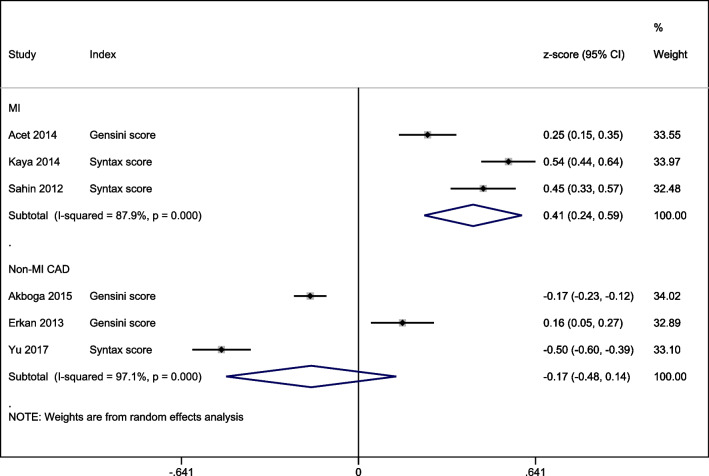
A forest graph showing the outcomes of a meta-analysis of z-scores of correlation coefficients between disease severity and bilirubin levels

In a meta-analysis of correlation coefficients, total bilirubin levels were significantly positively correlated with MI severity (*r* = 0.41 [95% CI: 0.24, 0.59]; *p* < 0.01) but were non-significantly inversely correlated with non-MI CAD severity (*r* =  − 0.17 [95% CI: − 0.48, 0.14]; *p* = 0.28; Fig. 5).

The female sex was significantly inversely associated with increasing quantiles of total bilirubin (− 8.16 [− 14.53, − 1.77]; *p* = 0.016) in MI patients. In MI patients, hemoglobin levels were positively associated with increasing quantiles of total bilirubin. None of the cardiovascular risk factors tested had a significant relationship with increasing total bilirubin quantiles (Table 2).

**Table 2:** Relationship (metaregression coefficient [95% CI]) of CAD prognostic factors with increasing quantiles of bilirubin

Factor	Non-MI CAD	MI
Age	0.174 [-1.148, 1.496]; *p* = 0.789	-0.488 [-2.832, 1.856]; *p* = 0.658
Females (%)	-2.450 [-11.350, 6.431]; *p* = 0.577	-8.164 [-14.531, -1.769]; *p* = 0.016
Body mass index	0.058 [-0.398, 0.519]; *p* = 0.797	-1.213 [-5.604, 3.178]; *p* = 0.444
Hypertension (%)	-0.966 [-8.020, 6.088]; *p* = 0.781	-1.261 [-10.397, 7.874]; *p* = 0.772
Dyslipidemia (%)	-1.682 [-8.649, 5.284]; *p* = 0.619	-2.629 [-25.826, 20.567]; *p* = 0.809
Diabetes mellitus (%)	-1.613 [-5.935, 2.707]; *p* = 0.451	1.637 [-11.572, 14.846]; *p* = 0.794
Smoking (%)	-1.421 [-6.014, 3.172]; *p* = 0.533	-0.709 [-7.872, 6.455]; *p* = 0.835
CVD history (%)	-1.210 [-5.969, 3.550]; *p* = 0.590	-0.290 [-4.775, 4.194]; *p* = 0.883
Total cholesterol	-1.582 [-17.828, 14.663]; *p* = 0.837	4.785 [-18.340, 27.909]; *p* = 0.640
Triglycerides	-5.881 [-25.141, 13.379]; *p* = 0.532	3–7.247 [-85.008, 70.513]; *p* = 0.827
High-density lipoprotein	1.550 [-2.562, 5.663]; *p* = 0.442	-0.711 [-23.041, 21.620]; *p* = 0.940
Low-density lipoprotein	0.991 [-6.170, 8.154]; *p* = 0.776	-0.809 [-3.912, 2.294]; *p* = 0.586
Hemoglobin		0.532 [0.103, 0.961]; *p* = 0.023
Serum creatinine	0.032 [-0.062, 0.126]; *p* = 0.472	0.012 [-0.050, 0.074]; *p* = 0.668
c-reactive protein	-0.014[-0.411, 0.385]; *p* = 0.939	0.676 [-0.844, 2.196]; *p* = 0.285
Alanine transaminase	0.740 [-0.959, 2.458]; *p* = 0.371	4.411 [-19.875, 28.698]; *p* = 0.60
Aspartate transaminase	0.737[-0.989, 2.463]; *p* = 0.384	27.278 [-21.803, 76.359]; *p* = 0.178
γ-glutamyltranspeptidase	3.204 [-4.485, 10.892]; *p* = 0.347	
Alkaline phosphatase	3.433 [-1.669, 8.536]; *p* = 0.162	
Statin use (%)	-6.804 [-20.256, 6.647]; *p* = 0.305	
ACEi/ARB use (%)	-0.905 [-10.049, 8.239]; *p* = 0.832	
Calcium channel blockers (%)	-2.829 [-17.783, 12.125]; *p* = 0.647	
Beta blockers use (%)	-6.034 [-20.581, 8.512]; *p* = 0.394	

## Discussion

This meta-analysis has found that bilirubin may have differing roles for MI and non-MI CAD patients as a) higher total bilirubin levels were associated with higher odds in MI patients but lower odds in non-MI CAD patients; b) total bilirubin levels were higher in severe MI patients but lower in severe non-MI CAD patients; and c) total bilirubin levels correlated positively with MI severity but correlated inversely with non-MI CAD severity. Thus, in general, higher bilirubin levels predicted poor outcomes in MI patients but better outcomes in non-MI CAD patients.

These outcomes are consistent with previously published meta-analyses on this topic. A meta-analysis found an inverse relationship between serum bilirubin levels and atherosclerosis in men [19]. Another meta-analysis has also reported a better prognosis for patients with arteriosclerotic diseases having higher serum total bilirubin levels [20]. Moreover, a meta-analysis found that higher bilirubin levels were associated with a reduced risk of first MI incidence [22]. On the other hand, meta-analyses have found a positive relationship between bilirubin levels and the incidence of MACE in patients with MI [22, 23].

In the third National Health and Nutrition Examination Survey (NHANES) that included 176,748,462 individuals from the general population, the serum bilirubin levels of the participants were 0.62 [0.61, 0.63] mg/dl [72]. This estimate is comparable to our pooled analysis that found serum total bilirubin levels to be 0.66 mg/dl [0.56, 0.75] in the general population. In NHANES survey, females had significantly lower bilirubin levels (0.52 vs 0.72 mg/dl) [72]. We have found an inverse relationship between the female percentage and increasing quantiles of bilirubin. Females may be more prone to adverse outcomes of CAD. A higher risk of MI or 1-month mortality is observed for women who underwent a coronary intervention [73, 74]. Women with CAD are also more vulnerable to the effects of diabetes [75, 76].

In the present study, it was not possible to perform a direct comparison of bilirubin levels between MI and non-MI CAD patients. In the pooled analysis, bilirubin levels did not differ appreciably between patients with MI, non-MI CAD, or healthy individuals. Importantly, high statistical heterogeneity observed in these analyses indicates wide variations within these three groups. Huang et al. [43] found significantly higher bilirubin levels in patients with MI in comparison with non-MI CAD patients. It has been reported that increased bilirubin levels decrease after the acute phase of MI [77]. Chung et al. [35] who found higher bilirubin levels to predict a worse prognosis in the acute phase of MI, did not find this one year after infarction, which coincides with the observation that heme oxygenase-1 (HO-1) activation is restricted to the acute phase only [77]. HO-1 is a rate-limiting enzyme. A strong correlation is observed between HO-1 activation and increased bilirubin levels in patients with MI or those undergoing percutaneous coronary intervention. However, the existence of HO-1 gene polymorphism in humans manifests inter-individual differences in the response of HO-1 to stress [78].

Currently, the precise role of bilirubin in the pathogenesis or prevention of CAD is not clear. Being an antioxidant, anti-apoptotic, and anti-inflammatory compound, it may participate in protecting or slowing the progression of atherosclerosis [71]. However, as observed in the present study, bilirubin offers a poor prognosis for MI but a good prognosis for non-MI CAD complicates the conceived role of bilirubin. It may be possible that adverse outcomes arising from the infarction itself may get associated with bilirubin which increases in such stressful events to reduce the harm but is unable to compensate for the overwhelming detrimental effects of infarction [65]. A U-shaped curve observed by many authors [50, 59, 79] to represent the relationship between the increasing quantiles of bilirubin levels and cardiovascular risk may also support this notion, as the highest bilirubin quantile may reflect acute stress-activated HO-1 activity.

Bilirubin is also found to show inverse relationships with several other diseases including diabetes, peripheral artery disease, Crohn’s disease, systemic lupus erythematosus, schizophrenia, and colorectal cancer [80]. Despite significant associations of bilirubin with cardiovascular outcomes observed in many studies, it is still not clear whether bilirubin has a true prognostic role or acts merely as a marker of disease. Mendelian randomization studies of UGT1A1 polymorphism associated with increased bilirubin levels found no association of bilirubin with stroke and CVD but found a negative association with type 2 diabetes [81, 82].

Present-day evidence on the role of bilirubin in the prognosis of CAD is also constrained by the use of non-standardized estimation methods, and reference values not tailored for sex, ethnicity, and age [80]. Whereas studies have shown that physiologically higher bilirubin levels predict better prognosis in non-MI CAD patients, this is not the case once MI develops. Our study further finds that the odds of an adverse outcome are higher with higher bilirubin levels in MI patients. Thus, more research is required to evaluate the role of bilirubin in non-MI and MI CAD patients and to clarify whether bilirubin has a real prognostic role or represents an epiphenomenon.

Differentiation of MI and non-MI CAD patients for meta-analyses is a strength of the present study. However, some limitations are also associated with this review. High statistical heterogeneity observed in many meta-analyses is an important consideration. Metaregression analyses did not indicate much about the sources of heterogeneity (I^2^). Other factors might had affected the heterogeneity e.g., HO-1 gene polymorphism causes inter-individual differences in the response of HO-1 to stress [78]. Another important limitation was the inclusion of relatively a smaller number of studies in the meta-analysis of SMD in bilirubin levels between lower and higher disease severity as well as in the meta-analysis of correlation coefficients between bilirubin levels and disease severity.

## Conclusion

This study found that higher bilirubin levels may predict the outcomes differently in non-MI CAD patients and MI patients as the odds of adverse outcomes with higher bilirubin levels were lower in non-MI CAD patients but were higher in MI patients. Moreover, bilirubin levels were lower in severe non-MI CAD patients but were higher in severe MI patients, and bilirubin levels correlated positively with MI severity but negatively with non-MI CAD severity. However, the presence of high statistical heterogeneity in most of the analyses warrants further studies with the power to delineate confounders to clarify the observed relationships of bilirubin with MI and non-MI CAD prognosis.

## Supplementary Information

**Additional file 1.** PRISMA_checklist.

**Additional file 2: Table S1.** Quality assessment of the included study with Newcastle-Ottawa Quality Assessment Scale. **Figure S1.** A funnel plot showing the outcomes of Begg’s test. **Appendix S1.** Literature search strategy.

## Data Availability

All relevant data specific to the present study are available with corresponding author.
