# Supplementary material for: Role of bilirubin in the prognosis of coronary artery disease and its relationship with cardiovascular risk factors: a meta-analysis
Source: BMC Cardiovasc Disord. 2022 Nov 2;22:458. doi: 10.1186/s12872-022-02899-w (PMC9632050; doi:10.1186/s12872-022-02899-w)
Supplement: Supplementary file 2 — Additional file 2: Table S1. Quality assessment of the included study with Newcastle-Ottawa Quality Assessment Scale. Figure S1. A funnel plot showing the outcomes of Begg’s test. Appendix S1. Literature search strategy. [file 12872_2022_2899_MOESM2_ESM.docx]

**Supporting information**

| **Table S1: Quality assessment of the included study with Newcastle-Ottawa Quality Assessment Scale** | | | | | | | | |
| --- | --- | --- | --- | --- | --- | --- | --- | --- |
| **Study** | **Represent-ativeness of exposed cohort** | **Selection of non-exposed cohort** | **Ascertain-ment of exposure** | **Demonstration that outcome of interest was not present at start of study** | **Comparability of cohorts based on design or analysis** | **Assessment of outcome** | **Was follow-up long enough for outcomes to occur** | **Adequacy of follow up completion of cohorts** |
| Acet 2014 | * |  | NA | NA | * | * |  |  |
| Agarwal 2015 | * |  | NA | NA | * | * |  |  |
| Akboga 2015 | * | * | NA | NA | * | * | * | * |
| Baumann 2016 | * |  | NA | NA | * | * |  |  |
| Canpolat 2013 |  |  | NA | NA | * | * |  |  |
| Celik 2014 | * |  | NA | NA | * | * |  |  |
| Chung 2016 | * |  | NA | NA | * | * | * | * |
| Ekblom 2010 | * | * | NA | NA | * | * |  |  |
| Elmohr 2021 | * |  | NA | NA | * | * |  |  |
| Erkan 2013 | * | * | NA | NA | * | * |  |  |
| Gul 2013 | * |  | NA | NA | * | * | * | * |
| Gullu 2005 |  |  | NA | NA | * | * |  |  |
| Hamur 2016 | * |  | NA | NA | * | * |  |  |
| Hopkins 1996 | * | * | NA | NA | * | * |  |  |
| Huang 2017 | * |  | NA | NA | * | * | * | * |
| Hunt 2016 | * | * | NA | NA | * | * |  |  |
| Kaya 2014 | * |  | NA | NA | * | * | * | * |
| Khalil 2019 | * |  | NA | NA | * | * |  |  |
| Kim 2014 | * |  | NA | NA | * | * | * | * |
| Kishimoto 2010 | * | * | NA | NA | * | * |  |  |
| Kuwano 2011 | * |  | NA | NA | * | * | * | * |
| Lai 2018 |  |  | NA | NA | * | * |  |  |
| Mahabadi 2014 |  |  | NA | NA | * | * | * | * |
| Miranda 2016 | * |  | NA | NA | * | * | * | * |
| Oda 2012 | * |  | NA | NA | * | * |  |  |
| Sahin 2012 | * |  | NA | NA | * | * |  |  |
| Schwertner 1994 | * |  | NA | NA | * | * |  |  |
| Song 2014 | * | * | NA | NA |  |  | * | * |
| Tanaka 2009 | * |  | NA | NA | * | * |  |  |
| Tatami 2014 | * |  | NA | NA | * | * |  |  |
| Troughton 2006 | * | * | NA | NA |  |  | * | * |
| Turfan 2012 | * |  | NA | NA | * | * |  |  |
| Tuxun 2020 | * |  | NA | NA |  |  | * | * |
| Wang 2016 |  |  | NA | NA |  |  | * | * |
| Wei 2012 | * |  | NA | NA | * | * |  |  |
| Xu 2018 | * |  | NA | NA | * | * |  |  |
| Xu 2019 |  |  | NA | NA | * | * | * | * |
| Yao 2015 | * |  | NA | NA | * | * | * | * |
| Yu 2017 | * |  | NA | NA | * | * | * | * |
| Zhang 2012 | * |  | NA | NA | * | * |  |  |
| Zhao 2020 | * |  | NA | NA | * | * | * | * |
| Zhu 2012 | * |  | NA | NA | * | * |  |  |
| Zhu 2016 | * | * | NA | NA | * | * |  |  |

Figure S1: A funnel plot showing the outcomes of Begg’s test.

**APPENDIX S1**

**Literature search strategy**

“Bilirubin” AND “Coronary artery disease” OR “CAD” OR “coronary heart disease” OR “CHD” AND “prognosis” OR “prognostic factor” AND “adverse cardiovascular outcome”

“Bilirubin” AND “Coronary artery disease” OR “CAD” OR “coronary heart disease” OR “CHD” AND “prognosis” OR “prognostic factor” OR “association” OR “correlation” AND “adverse cardiovascular outcome”

“Bilirubin” AND “Coronary artery disease” OR “CAD” OR “coronary heart disease” OR “CHD” “acute coronary syndrome” OR “ACS” OR “ischemic heart disease” OR “IHD” OR “angina” OR “myocardial infarction” OR “MI” OR “percutaneous coronary intervention” OR “PCI” AND “prognosis” OR “prognostic factor” OR “association” OR “correlation” AND “adverse cardiovascular outcome”

Note: Same strategy was used for each database on multiple trial basis.
